# Current trends in gene recovery mediated by the CRISPR-Cas system

**DOI:** 10.1038/s12276-020-0466-1

**Published:** 2020-07-10

**Authors:** Hyeon-Ki Jang, Beomjong Song, Gue-Ho Hwang, Sangsu Bae

**Affiliations:** 1 Department of Chemistry and Research Institute for Convergence of Basic Sciences, Hanyang University, Seoul, 04763 South Korea; 2 International Research Center for Neurointelligence (WPI-IRCN), The University of Tokyo, Hongo, Bunkyo-ku, Tokyo 113-0033 Japan

**Keywords:** Genetic engineering, Targeted gene repair, Genetics research, Genetic engineering, Targeted gene repair

## Abstract

The CRISPR-Cas system has undoubtedly revolutionized the genome editing field, enabling targeted gene disruption, regulation, and recovery in a guide RNA-specific manner. In this review, we focus on currently available gene recovery strategies that use CRISPR nucleases, particularly for the treatment of genetic disorders. Through the action of DNA repair mechanisms, CRISPR-mediated DNA cleavage at a genomic target can shift the reading frame to correct abnormal frameshifts, whereas DNA cleavage at two sites, which can induce large deletions or inversions, can correct structural abnormalities in DNA. Homology-mediated or homology-independent gene recovery strategies that require donor DNAs have been developed and widely applied to precisely correct mutated sequences in genes of interest. In contrast to the DNA cleavage-mediated gene correction methods listed above, base-editing tools enable base conversion in the absence of donor DNAs. In addition, CRISPR-associated transposases have been harnessed to generate a targeted knockin, and prime editors have been developed to edit tens of nucleotides in cells. Here, we introduce currently developed gene recovery strategies and discuss the pros and cons of each.

## Experimental & Molecular Medicine: Genetic disease: Gene editing for recovery

The CRISPR-Cas gene editing system, which relies on small RNA molecules to guide a gene-editing enzyme to specific locations on DNA, is being developed as an effective tool for correcting genetic disorders. Researchers in South Korea led by Sangsu Bae at Hanyang University in South Korea, review recent progress towards such “gene recovery” procedures. The possibilities range from correcting mutations at the level of a single base in the base sequence of DNA, to deleting, inverting or inserting large sections of DNA to correct major structural abnormalities. The authors discuss the pros and cons of different procedures, including CRISPR-Cas nucleases, base editors, and prime editors. They expect current laboratory animal investigations will lead to a new era in human genetic medicine, yielding treatments for genetic diseases that cannot currently be treated with drugs.

## Introduction

Human genetic disorders, often associated with severe pathological phenotypes, are caused by genomic aberrations such as gene mutations and chromosomal abnormalities. Therefore, a reliable therapeutic method for gene recovery would be quite valuable. Previously, exogenous delivery of therapeutic normal genes via viral vehicles, such as adenoviruses, adeno-associated viruses (AAVs), and lentiviruses, has been tried as a means of providing the normal function of the inactivated/disrupted gene^1^. Although such gene therapy methods have produced successful therapeutic results, this general approach has potential limitations. For example, the exogenous gene is constitutively expressed, unaffected by the native chromatin structure of the endogenous locus, at a level that differs from that of the endogenous gene^2^. Furthermore, the mutated endogenous gene, which is malfunctional and potentially cytotoxic, might still be transcribed.

Precise correction of the endogenous gene of interest is a strongly desirable alternative for gene recovery. Programmable nucleases, which include zinc-finger nucleases (ZFNs)^3^, transcription activator-like effector nucleases (TALENs)^4^, and clustered regularly interspaced short palindromic repeat (CRISPR)-CRISPR-associated (Cas) endonucleases^5–7^, enable target-specific DNA cleavage and gene editing. CRISPR-mediated gene-editing technologies are now overwhelmingly the method of choice because of their ease of handling and low cost. Since CRISPR nucleases were first harnessed for generating site-specific DNA cleavage in the human genome, new CRISPR-based gene-editing tools, including base-editing technologies, have been developed. The ability to correct endogenous genes in a targeted and predictable manner using such tools has undoubtedly revolutionized gene-based drug development as well as basic research. In this review, we introduce current trends in CRISPR-mediated gene correction and rescue strategies and describe the pros and cons of each tool.

## DNA repair pathways induced by CRISPR-mediated DNA cleavage in eukaryotic cells

The type II CRISPR-Cas9 and type V CRISPR-Cas12a (also known as Cpf1) endonucleases are targeted to specific genomic sites by associated guide RNAs^8,9^ and can be used to generate site-specific DNA cleavage in various cell types and organisms, including humans. Typically, researchers use one piece of single guide RNA (sgRNA), which includes a spacer region complementary to the target DNA and a region that binds to the endonuclease. The target DNA sequence recognized by the guide RNA must be associated with a nuclease-specific protospacer adjacent motif (PAM), which is recognized directly by the endonuclease.

**Fig. 1:**
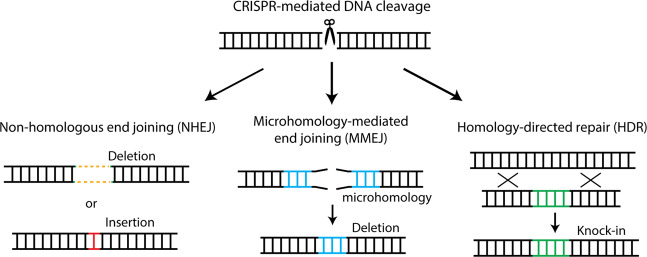
Schematic of the cell’s own repair processes. They include the non-homologous end joining (NHEJ) pathway, the microhomology-mediated end joining (MMEJ) pathway, and the homology-directed repair (HDR) pathway.

The chromosomal double-strand breaks (DSBs) produced by these nucleases are typically repaired by the cell’s own repair processes, such as the non-homologous end joining (NHEJ) pathway, the homology-directed repair (HDR) pathway, or an alternative KU-independent process such as the microhomology-mediated end joining (MMEJ) pathway^10^ (Fig. 1). DSBs are ligated without a homologous template during the NHEJ process, which frequently leads to small nucleotide insertions and deletions (indels) at the cleavage site. In the presence of a donor DNA template, HDR precisely rejoins the DSB ends based on the donor DNA sequence, which results in precise gene corrections or knockins. The MMEJ pathway is an alternative NHEJ pathway that involves annealing between identical microhomologous sequences (>2 bp) flanking the DSB. Hence, MMEJ causes sequence-dependent deletions according to the microhomologous sequences that flank the cleavage site. On the basis of these various repair pathways, researchers have established precise endogenous gene recovery strategies in human cells for treating different genetic diseases.

## Gene recovery strategies in the absence of donor DNA

### Frameshift- and deletion-mediated gene recovery involving one guide RNA

**Fig. 2:**
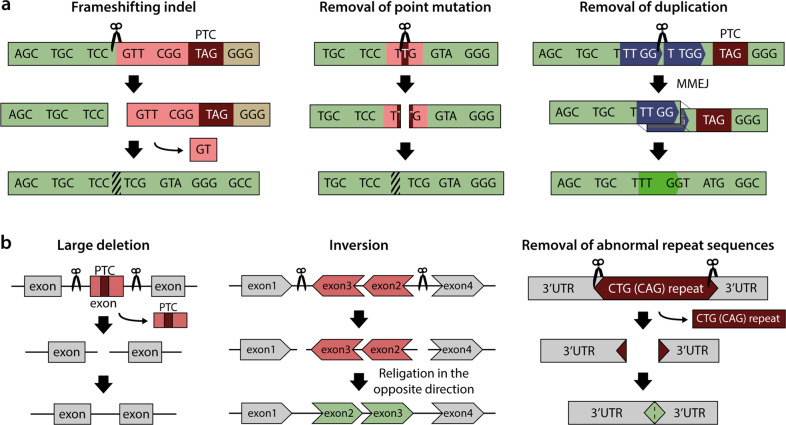
Gene recovery strategies in the absence of donor DNA. **a** Gene recovery methods with one guide RNA. **b** Gene recovery methods with dual guide RNAs. PTC premature termination codon.

NHEJ, a dominant repair pathway in mammalian cells that is active throughout the cell cycle^10^, is typically used for gene disruption or knockout via induction of indels^11,12^. Alternatively, however, NHEJ-mediated indels can be effectively used for genetic disease treatment if they induce a desired frameshift or delete a point mutation (Fig. 2a). For example, in Duchenne muscular dystrophy (DMD) models, premature stop codons induced by deletion of exon 44 were corrected by the introduction of Cas9-mediated frameshifting indels at a nearby location^13,14^. Additionally, point mutations that lead to aberrant splicing in the *DMD* gene or in the *Hemoglobin B (HBB)* gene, which cause β-thalassemia, were removed by the introduction of Cas9- or Cas12a-mediated indels^15,16^.

Similar to the NHEJ-mediated gene recovery strategy, MMEJ-mediated deletions can also be used to remove disease-causing mutations **(**Fig. 2a). An 8-bp duplication in exon 1 of the *TCAP* gene that causes limb-girdle muscular dystrophy type 2 G (LGMD2G) was deleted precisely in patient-derived induced pluripotent stem cells (iPSCs) and myoblasts differentiated from the iPS cells via the MMEJ pathway^17^. Likewise, MMEJ was utilized to remove a 16-bp microduplication in exon 15 of the HPS1 gene in B lymphocytes that causes Hermansky–Pudlak syndrome type-1 (HPS1).

In addition, several programs, such as Microhomology predictor^18^, inDelPhi^19^, DeepSpCas9^20^, and DeepCpf1^21^, have been developed to predict gene-editing efficiencies and/or editing outcomes after CRISPR treatment. These resources should accelerate the use of frameshift- and small deletion-mediated gene recovery strategies.

### Large deletion- or inversion-mediated gene recovery involving two guide RNAs

CRISPR nuclease target sites can be changed simply by altering the sgRNA sequence; multiple DNA cleavages are easily obtained by using two or more sgRNAs simultaneously. Therefore, researchers can generate a large deletion or inversion of a gene of interest by using dual sgRNAs (Fig. 2b). Because CRISPR-mediated DNA cleavage is typically accompanied by indel formation at target sites, introns may be more suitable than exons as target sites in such approaches. For example, the abnormal CTG repeat in the 3′ untranslated region of the *DMPK* gene that results in myotonic dystrophy type-1 was successfully excised in patient-derived iPSCs and muscle cells by treatment with Cas9 and dual sgRNAs^22,23^. Additionally, mutation-carrying exons were excised by using dual sgRNAs in myoblasts derived from a DMD mouse model and in keratinocytes derived from patients with recessive dystrophic epidermolysis bullosa (RDEB); this approach restored gene function^24,25^. Cas9 with dual sgRNAs can induce not only large deletions but also inversions, which occur when the excised gene fragment is religated in the opposite direction at the same locus. A large inversion in the *FVIII* gene associated with hemophilia A was reoriented by using Cas9 with dual sgRNAs in hemophilia A patient-derived iPSCs^26^.

## Gene recovery strategies involving donor DNAs

### Precise HDR-mediated gene correction

**Fig. 3:**
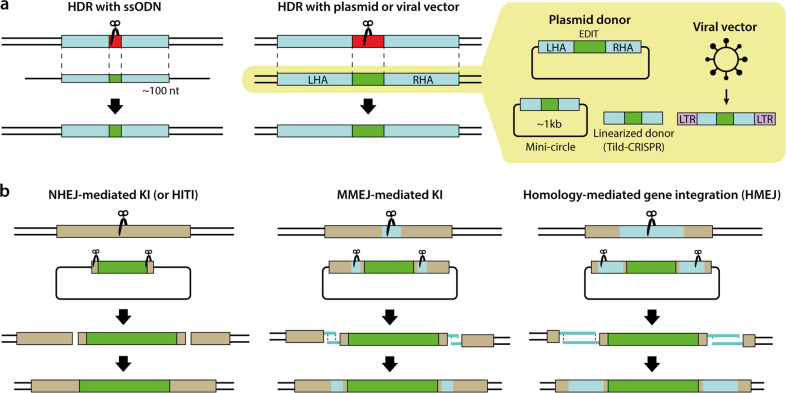
Gene recovery strategies involving donor DNAs. **a** HDR-mediated gene correction rescue methods. **b** Homology-independent gene recovery methods. LHA left homology arm, RHA right homology arm, LTR long terminal repeat.

HDR-mediated gene correction is the most popular strategy for gene recovery because the genetic defect is corrected to exactly match the DNA donor template. To date, HDR-mediated gene correction has been widely harnessed to treat various genetic diseases, including sickle cell disease^27–29^, ß-thalassemia^30–34^, hemophilia A/B^35–38^, and DMD^14,39,40^, in cells or organisms. The DNA donors for HDR have been provided in various forms, including single-stranded DNA oligonucleotides (ssODNs), DNA plasmids or viral vectors, as discussed below^41^ (Fig. 3a).

For correction of one or a few mutations, an ssODN donor template is preferable because of its low cost and the relatively high associated editing efficacy. The relatively small size of ssODNs (90 ~ 200 nt) is advantageous for the synthesis and delivery of the donor template. Furthermore, ssODNs show a low level of chromosomal integration compared to double-stranded DNA oligonucleotides (dsODNs)^42^. A dominant cataract disorder in mice was the first genetic disease for which the ssODN strategy was used to correct the causative mutation, a 1-bp deletion in exon 3 of the *Crygc* gene^43^ that leads to the production of truncated γC-crystallin^44^. Other diseases associated with single base substitutions, such as DMD (*DMD* gene)^39,40^, achondroplasia (*FGFR3* gene)^45^, Alzheimer’s disease (*PSEN2* gene)^46^, retinitis pigmentosa (*Pde6b* gene)^47^, sickle cell disease (*HBB* gene)^48^, and hemophilia B (*HBB* gene)^37^, have also been targeted with ssODN-templated gene correction. Additionally, a 4-bp deletion in the *HBB* gene (causing β-thalassemia)^32,33^ and a 4-bp insertion in the *FANCF* gene (causing Fanconi anemia)^49^ were corrected exactly by the ssODN-templated HDR strategy.

The ssODN strategy is convenient for gene correction at the cellular level but is not easily applied for in vivo corrections in organisms such as mice due to the lack of a reliable delivery method. For example, ssODNs cannot be carried by viral vehicles, in contrast with DNA plasmids. Hence, donor templates for in vivo applications are typically prepared as plasmids carrying the desired sequence flanked by long homology arms (~800 bp) (Fig. 3b). The donor plasmid template resembles an endogenous template (i.e., the opposite allele) in the homologous recombination process. HDR-mediated genome editing with a plasmid donor can precisely replace a long stretch of nucleotides or insert large constructs, such as a sequence encoding a tagged protein, at a specific locus. Cystic fibrosis was the first genetic disease for which HDR-mediated gene correction with a donor plasmid was performed in patient-derived intestinal stem cells carrying a homozygous 3-bp deletion in exon 11 of the *CFTR* gene^50^. The donor plasmid contained a puromycin resistance gene in addition to the wild-type *CFTR* sequence for effective selection of knockin cells. Indeed, the availability of selection markers, including drug resistance genes or genes encoding fluorescent proteins, is one of the benefits of using a plasmid donor versus an ssODN donor. By using HDR with a plasmid carrying donor DNA, various mutation patterns have been corrected, including small-sized mutations in the *HBB* gene (associated with β-thalassemia)^30,31,34^ and the *RPGR* gene (associated with retinitis pigmentosa)^51^, as well as large-sized mutations corrected by the precise knockin of a large DNA fragment from the *FVIII* gene (associated with hemophilia A)^38^ and from the *DMD* gene (associated with DMD)^13^.

However, the efficiency of plasmid-templated HDR is typically lower than that of ssODN-templated HDR. To enhance the editing efficiency of plasmid-templated HDR, some modifications have been adopted. A minicircle plasmid (~1 kb), the size of which was minimized by removal of the bacterial backbone sequence, was utilized as a donor template to increase transfection efficiency^52^. Additionally, linearized templates that were prepared by PCR amplification or restriction enzyme-mediated digestion were associated with increased editing efficiencies in various cell types; this method was termed targeted integration with linearized dsDNA-CRISPR (Tild-CRISPR)^53^.

To maximize donor plasmid delivery into the cell nucleus, recent studies used viral vectors, including an integrase-defective lentiviral vector, adenoviral vector, or adeno-associated viral vector, as the donor template. Genetic mutations associated with SCD^27–29^, hemophilia B^35,36^, or RDEB^54^ were corrected by Cas9-mediated HDR using a viral vector donor. Delivery of an AAV6 donor along with Cas9 RNPs to SCD patient-derived hematopoietic stem and progenitor cells (HSPCs) resulted in successful targeted gene correction at 19% efficiency^27^. The combination of Cas9 RNPs and an AAV6 donor template has been reported to be a powerful tool for gene correction in HSPCs, T cells, and iPSCs, resulting in precise editing efficiencies of ~60%^29,55,56^. Notably, AAV donors can be used as an alternative to plasmid donors, especially in some cell types that show poor HDR editing efficiency or cytotoxicity when transfected with plasmids.

### Homology-independent gene recovery

Despite the precision of HDR-mediated gene correction, it is not the method of choice in all circumstances. This method frequently shows low editing efficacy, and its utility is limited in non-dividing or fully differentiated cells, because HDR is active only in the late S and G2 phases of the cell cycle^57^. In contrast, gene expression cassettes can be integrated via the NHEJ-mediated knockin method regardless of the cell cycle phase^58^ (Fig. 3b). The representative example of this method is homology-independent targeted integration (HITI)^59^. HITI employs donor plasmids that lack homology arms but include Cas9 cleavage sites flanking the donor sequence. Therefore, Cas9 nucleases cut both the genomic target sequence and the donor plasmid, after which the cleaved donor DNA can be incorporated into the target gene. One remarkable property of HITI is its high accuracy even in fully differentiated cells such as neurons^60^, which can be achieved by repeating Cas9-mediated cleavage until the donor DNA is inserted in the desired orientation^59^. This property of HITI makes it a promising method for curing genetic diseases by gene replacement. Royal College of Surgeons (RCS) rats, an animal model of retinitis pigmentosa caused by deletion in the *Mertk* gene, have morphological changes in the degenerating photoreceptor outer nuclear layer (ONL)^61^. Injection of HITI-AAV vectors in the subretinal space in the eyes of RCS rats significantly increased *Mertk* mRNA levels and preserved ONL thickness^59^. Consistent with this, the MERTK protein was observed in the eyes, and electroretinography tests showed improved eye function^59^.

Similar to NHEJ, MMEJ can also mediate knockin of a large gene construct. In a technique known as CRIS-PITCh, CRISPR nucleases are used to cleave both genomic and donor DNA at sites with microhomology, resulting in precise integration into the target chromosome^62^ (Fig. 3b). CRIS-PITCh requires three sgRNAs, and the CRIS-PITCh vector must include two different sgRNA target sites. A recent study showed the potential for CRIS-PITCh in gene replacement therapy. Hydrodynamic injection of Cas9-expressing and *Fah*-MMEJ constructs into *Fah*^*−/−*^ mouse livers, which are a model of hereditary tyrosinemia type I (HTI) caused by a deficiency in fumarylacetoacetate hydrolase due to mutation of the *Fah* gene, resulted in correction of the *Fah* gene in hepatocytes, which alleviated symptoms such as body weight loss and liver damage as well as prolonged the lives of the *Fah*-corrected mice^63^.

To maximize the editing efficacy relative to that seen with the HDR-, NHEJ-, and MMEJ-mediated knockin strategies described above, a new combination strategy using long homology arms (~800 bp) was developed, referred to as homology-mediated gene integration (HMEJ)^64^ (Fig. 3b). In this technique, the HMEJ construct contains homology arms similar to those used for HDR as well as CRISPR targets flanking the donor DNA similar to those used for NHEJ, enabling HMEJ to be mediated by either NHEJ or HDR depending on the cell type. In non-dividing cells such as astrocytes and neurons, HMEJ resulted in a gene-editing efficiency that was comparable with that of NHEJ- and MMEJ-based methods. Interestingly, an HDR inhibitor (caffeine) decreased HMEJ-mediated gene knockin in mouse embryonic stem (ES) cells but not in neurons, whereas an NHEJ inhibitor (Scr7 or Nu7026) decreased the HMEJ-mediated gene-editing efficiency in neurons but not in mouse ES cells^64^. This dual nature of HMEJ represents a ray of hope in gene therapy. Indeed, HMEJ was effective for treating HTI. Hydrodynamic injection of HMEJ constructs into *Fah*^*−/−*^ mice resulted in greater proliferation of normal hepatocytes than the MMEJ strategy^65^.

## Gene recovery by base editors without DNA DSB generation

Mutation of a single nucleotide in a gene, which can induce an amino acid substitution in the encoded protein (missense mutation) or truncation of the protein (nonsense mutation), is the main cause of genetic diseases (>58% of the entries in the ClinVar database)^66^. Although the single mutated nucleotide can be precisely repaired through HDR, the low editing efficiency and the restriction to non-dividing cells of this method obstruct its therapeutic application. In addition, recent studies have warned that DNA DSBs can lead to a p53-mediated DNA damage response^67,68^ and can frequently cause unexpected large deletions^69,70^. Therefore, an alternative gene correction method that does not generate DNA DSBs is required. CRISPR-based base-editing tools, including cytosine and adenine base editors (CBEs and ABEs), have recently been developed for highly efficient single nucleotide correction, which occurs without donor DNA but does not generate DNA DSBs^71,72^.

### Cytosine base editors (CBEs)

**Fig. 4:**
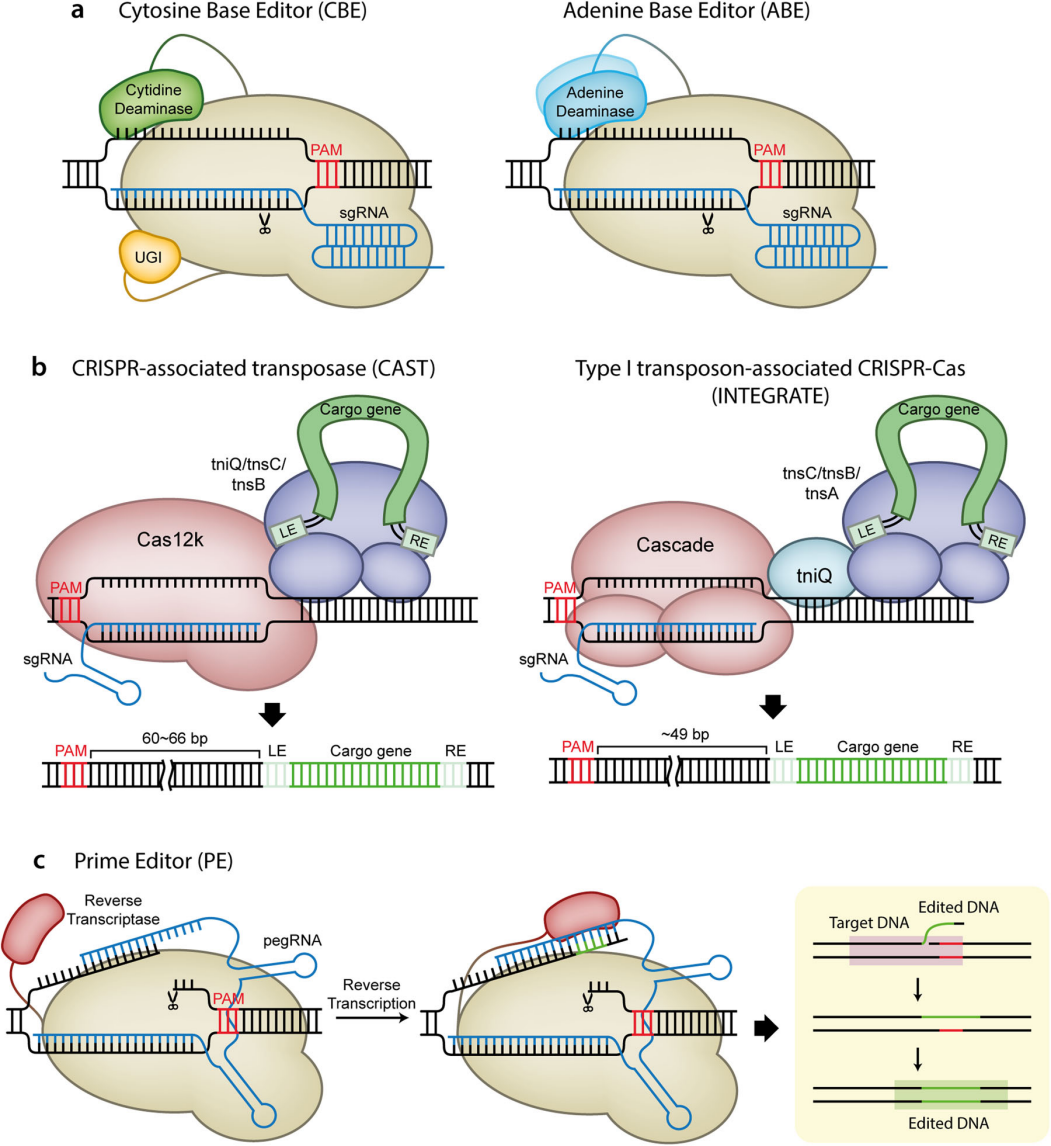
Gene recovery strategies in the absence of DNA DSB generation. **a** Schematic of a cytosine base editor (CBE), an adenine base editor (ABE). **b** Schematic of the insertion of transposable elements by guide RNA-assisted targeting (INTEGRATE) with a type I transposon-associated CRISPR-Cas system and of RNA-guided DNA insertion with a CRISPR-associated transposase (CAST). LE transposon left end, RE transposon right end. **c** Schematic of a prime editor (PE) and its working mechanism. Reverse transcriptases in PEs copy the information in the pegRNAs into DNA target sites.

The optimized forms of CBEs (BE3 and TARGET-AID) are composed of three proteins: a partially inactive Cas9 variant that exhibits nickase activity (nCas9) fused to a cytidine deaminase such as rAPOBEC1 or pmCDA1 and a uracil DNA glycosylase inhibitor (UGI)^71,73^ **(**Fig. 4). The CBE initially replaces a cytosine in the nontarget strand with uracil, after which the guanine in the target strand that was previously paired with cytosine is replaced with adenine by the cell’s repair mechanism. Finally, the uracil is replaced with thymine, thereby generating a T–A pair in place of the previous C–G pair^71^. UGI prevents the base excision repair process that removes uracil from the nontarget strand, enhancing the C-to-T conversion efficiency.

Subsequent studies led to the development of several CBE variants with improved editing efficiency. The editing window of BE3 was narrowed by altering the sequences linking nCas9 to the cytidine deaminase^74^ or by APOBEC1 mutation^75^, and BE3 expression was enhanced in mammalian cells by optimizing the nCas9-encoding sequences^76^. Adding a second UGI on BE3 (BE4) decreased the formation of undesired byproducts of editing^77^. BE4 was further improved by adopting nuclear localization signal (NLS) sequences and optimized codons (BE4max) and by employing APOBEC homologs (AncBE4max)^78^.

CBEs show great promise for the treatment of genetic diseases because many preliminary studies have shown tangible results. For example, nucleofection of astrocytes with BE3 constructs converted the apolipoprotein E gene 4 (*APOE4*), the most common genetic risk factor for late-onset Alzheimer’s disease, to *APE3r*, a less risky form^71^. The p53 gene mutation, which causes many types of cancers, was corrected by BE3 in human breast cancer cells (HCC1954)^71^. A nonsense mutation in the superoxide dismutase 1 (*SOD1*) gene was induced by BE3 in amyotrophic lateral sclerosis (ALS) model mice, prolonging survival^79^. The *HBB* gene mutation that causes β-thalassemia was corrected by BE3 or its variant YEE-BE3 in skin fibroblasts, human embryos, and blastomeres^80^. The *FBN1* mutation, a cause of Marfan syndrome^81^, was repaired in human embryos using YE1-BE3 or YEE-BE3^82^.

A successful CRISPR-based gene correction strategy requires that an appropriate PAM be located near the mutation of interest. The most commonly used Cas9 nuclease, SpCas9 from *Streptococcus pyogenes*, recognizes a 5′-NGG-3′ PAM downstream of the target, but such motifs are not always present. This limitation can be overcome by swapping the Cas9 nickase with other nickases preferring different PAM sequences. nSaKKH-BE3, which adopts mutated SaCas9 derived from *Staphylococcus aureus*, recognizes alternative PAM sequences (5′-NNNRRT-3′) and was used to correct the *Pah* gene mutation in the hepatocytes of phenylketonuria model mice^83^.

### Adenine base editors (ABEs)

Analogous to CBEs, ABEs replace adenines in the nontarget strand with inosines, generating I-T pairs. Because inosine preferentially base pairs with cytosine over thymine, DNA repair machinery converts the I-T pairs to I-C pairs and finally to G-C pairs^72^. ABEs differ from CBEs in that they contain deoxyadenosine deaminase instead of cytidine deaminase **(**Fig. 4**)**. Because a natural deoxyadenosine deaminase is not known to exist, directed evolution for protein engineering was used to convert a transfer RNA adenosine deaminase, TadA, to a DNA deoxyadenosine deaminase, referred to as TadA*, which was then fused to a nCas9 to create ABE7.10^72^. This initially optimized ABE was further improved by optimizing codons and adding an NLS sequence (ABEmax)^78^. Recently, various ABEmax variants, based on different Cas9 variants, were developed (VRQR-ABEmax, VRER-ABEmax, xABEmax, NG-ABEmax, SaABEmax, and SaKKH-ABEmax); these versions expand the target sites of ABEs^84^.

Similar to the situation with CBEs, there have been many efforts to apply ABEs to gene therapy. For example, the expression of the *HBG1* and *HBG2* genes in adults is thought to alleviate the symptoms of β-globin-related blood diseases, and ABE7.10 and ABEmax were successfully used to induce the desired mutation in the *HBG1* and *HBG2* promoters in HEK293T cells^72,78^. The mutation in the *HFE* gene causing hereditary hemochromatosis was corrected by ABE7.10 in an immortalized lymphoblastoid cell line^72^. In addition, by converting a premature stop codon in *DMD* to a Glu codon, ABE7.10 restored dystrophin expression in myofibers of DMD model mice^85^. In HTI model mice, targeting RA6.3 (an improved ABE6.3 variant) to the *Fah* gene corrected the HTI-associated mutation and alleviated disease symptoms^86^. In another example, a mutation in the *COL7A1* gene causing recessive RDEB^87,88^ was corrected by ABEmax in primary fibroblasts and iPSCs^89^. Furthermore, CjABE, an ABE that contains catalytically impaired Cas9 from *Campylobacter jejuni*, was used to correct a brain tumor-associated mutation in the *TRET* gene in primary glioblastomas^90^. Finally, CRISPR-pass is a general ABE-based method of inducing premature stop codon read-through. When used in fibroblasts from xeroderma pigmentosum complement group C (XPC) patients, this technique led to read-through of the disease-associated premature stop codon, thereby generating functional protein^91^.

## New gene recovery strategies: CRISPR-associated transposase and prime editors

Recently, it was reported that several Tn7-like transposons are associated with CRISPR-Cas systems^92,93^; this finding has led to the development of new gene-editing tools (Fig. 4). One approach is to use a CRISPR-associated transposase from the cyanobacterium *Scytonema hofmanni* (ShCAST); this transposase is made up of Tn7-like transposase subunits and a type V Cas12k^94^. The ShCAST complex is recruited to the target site in an RNA-guided manner due to the type V Cas12k and unidirectionally inserts the cargo genes of the donor plasmid into the target site via its transposase subunits. The other tool is insertion of transposable elements by guide RNA-assisted targeting (INTEGRATE) based on a type I transposon-associated CRISPR-Cas system^95^. INTEGRATE consists of a CRISPR-associated complex for antiviral defense (Cascade) complex (Cas6, Cas7, and Cas8), which directs the editing machinery to the target site, and Tn7-like transposase subunits for cargo gene insertion into the target site. INTEGRATE differs from ShCAST in that it additionally requires the Tn7 transposition protein tnsA and can insert the cargo gene into the target site bidirectionally. Although both ShCAST and INTEGRATE have been tested only in *Escherichia coli* to date^94,95^, it is expected that they can be employed as great alternatives to NHEJ for gene knockin therapy through subsequent improvement, which will enable them to function in eukaryotic cells.

Another new approach involves the use of a prime editor (PE), which is composed of a reverse transcriptase (RT) and a Cas9 nickase, and a unique “prime editing” guide RNA (pegRNA)^96^ (Fig. 4). A PE that is recruited to the target site by a pegRNA generates a nick in the PAM-containing strand. Because the pegRNA has a template sequence at its 3’ end, the RT enzyme of a PE can copy the information from the template to the 3’ end of the nicked strand of DNA. This in situ synthesized donor-templated repair of DNA enables PEs to precisely induce substitutions, including transversion and transition, as well as indels, which cannot be achieved by conventional base editors^71–73,96^. In addition, PEs showed low off-target editing effects and generated negligible byproduct mutations, suggesting that PEs shows promise in medical applications^96^. Indeed, PEs effectively corrected the mutations in the *HBB* gene and *HEXA* gene in HEK293T cells, which cause sickle cell disease and Tay-Sachs disease, respectively^96^. Furthermore, a recent study revealed that a plant prime editor (PPE), an optimized version of PEs for plant cells, can induce various substitutions and indels efficiently in rice and wheat protoplasts^97^, suggesting that PEs can be applied to diverse systems, including plants and animals.

## Conclusion

In this review, we have focused on CRISPR nuclease-mediated gene recovery strategies rather than methods for gene knockout or gene regulation such as gene inhibition (CRISPRi) or activation (CRISPRa)^98^. Gene correction technologies have been rapidly developed and widely tested for the treatment of many genetic diseases (Table 1). Gene correction based on DNA cleavage in the absence of donor DNA is relatively simple if frameshifting will lead to gene recovery but is of limited value for correcting many types of mutations, such as substitutions. For such purposes, HDR-mediated gene correction is the most precise and well characterized method, but its usefulness is limited because of its low efficiency, lack of activity in non-dividing cells, and need for donor DNA. Although NHEJ- or MMEJ-mediated gene correction enable gene knockin even in non-dividing cells with higher efficacy than HDR, these methods still require donor DNA and are typically less precise than HDR. Furthermore, recent studies have reported unexpected outcomes, including large deletions and chromosomal translocations, after DNA cleavage^69,70^; such results warn against the use of DNA cleavage-mediated gene recovery strategies.

**Table 1:** Current gene correction treatments for many genetic diseases.

Disease	Target gene	Type of mutation	Gene recovery strategy
Achondroplasia	*Fgfr3*	c.1120 G > A (p.G374R)	HDR (ssODN)^45^
Alzheimer’s disease (AD)	*PSEN2*	c.422 A > T (p.N141I)	HDR (ssODN)^46^
	*APOE*	ApoE4 Arg158^a^	CBE^71^
Amyotrophic lateral sclerosis (ALS)	*SOD1*	c.281 G > C (G94A) (G93A transgenic mouse)	CBE (KO of mutant *SOD1)*^79^
β-thalassemia	*HBB*	c.93-21 G > A or c.316-197 C > T (inducing an aberrant splice)	Indels to remove a point mutation^16^
		c.126_129delCTTT (CD 41/42 (-CTTT))	HDR (ssODN)^32,33^
			HDR (donor plasmid)^30,34^
		c.654 C > T	HDR (donor plasmid)^31^
		g.-28A > G	CBE^80^
β-globin-related disease	*HBG1*	g.-175T or g.-198T^a^	ABE^72,78^
Cancer	*TP53*	c.488 A > G (p.Y163C)	CBE^71^
Cataract	*Crygc*	c.461delG (at exon 3)	HDR (ssODN or w/o donor)^43b^
Cystic fibrosis	*CFTR*	c.1521_1523delCTT (p.F508del)	HDR (donor plasmid)^50^
Congenital disorder of glycosylation (CDG) type-1 f	*MPDU1*	c.356 T > C (p.L119P)	CBE^78^
Chronic pain	*Scn9a*	c.689-1 C^a^	CBE^78^
Duchenne muscular dystrophy (DMD)	*DMD*	c.2983 C > T (at exon 23, p.Q995X, mdx mouse)	Large deletion using dual sgRNAs^24^
			HDR (ssODN)^39,40^
			HDR (Adv donor)^39^
		Deletion of exon 44 introducing a PTC into exon 45	Frame-fitting indels^13,14^
			Indels for skipping exon 45^13,14^
			HDR (donor plasmid)^14^
		Gross deletion of exons 48-50 introducing a PTC into exon 51	Indels to disrupt a splice acceptor at exon 51 for skipping exon 51^15^
		c.6913-4037 T > G (a cryptic splice acceptor at intron 47)	Indels to remove the cryptic splice acceptor^15^
		Gross duplication of exons 55-59	Large deletion using dual sgRNAs^15^
		c.2611 C > T (p.Q871X)	ABE^85^
Epidermolysis bullosa simplex (EBS)	*KRT14*	c.1231 G > A (at exon 6)	HDR (donor plasmid)^52^
Fanconi anemia	*FANCF*	c.828InsTAAA	HDR (ssODN)^49^
Hemophilia A (HA)	*FVIII*	Gross chromosomal inversion of 140-kbp or 600-kbp involving introns 1 and 22	Large inversion using dual sgRNAs^26^
		Gross deletion of 94,172 bp from exon 8 to intron 22	HDR (donor plasmid)^38^
Hemophilia B (HB)	*FIX*	c.1111 T > G (p.Y371D, mouse)	HDR (ssODN or donor plasmid)^37^
		c.1477 G > A (p.Q418G, dog)	HDR (AAV donor)^35^
			HDR (Adv donor)^35^
		c.1136 G > A (p.R379Q) (R333Q transgenic mouse)	HDR (Adv donor)^36^
Hereditary tyrosinemia type I (HTI)	*Fah*	Insertion of a neomycin selection cassette at exon 5 (*Fah*^*∆exon5*^ mouse)	MMEJ-mediated KI^63^
			HMEJ-mediated KI^65^
		c.706 G > A (exon 8 skipping, *Fah5981SB* mouse)	ABE^86^
Hermansky–Pudlak syndrome	*HPS1*	c.1472_1487dup (16-bp duplication)	MMEJ^17,19^
Hereditary hemochromatosis (HHC)	*HFE*	c.845 G > A (p.C282Y)	ABE^72^
Limb-girdle muscular dystrophy (LGMD)	*TCAP*	c.26_33dup (8-bp duplication)	MMEJ^17^
Marfan syndrome	*FBN1*	c.7498 T > C	CBE^82^
Menkes disease	*ATP7A*	c.6913_6917dupCTTAT	MMEJ^19^
Myotonic dystrophy type-1 (DM1)	*DMPK*	CTG repeat expansion in the 3’UTR	Large deletion using dual sgRNAs^22,23^
Phenylketonuria (PKU)	*Pah*	c.835 T > C (p.F263S)	CBE^83^
Retinitis pigmentosa	*Pde6b*	c.1041 C > A (p.Y347X)	HDR (ssODN)^47^
	*RPGR*	c.1685_1686del (at exon 14)	HDR (donor plasmid)^51^
	*Mertk*	Gross deletion of 1.9 kbp from intron 1 to exon 2	NHEJ-mediated KI (HITI)^59^
Recessive dystrophic epidermolysis bullosa (RDEB)	*COL7A1*	c.189delG (at exon 2)	HDR (IDLV donor)^54^
		c.6527insC (at exon 80)	Large deletion using dual sgRNAs^25^
		c.553 C > T (p.R185X) or c.1573 C > T (p.R525X)	ABE^89^
Sickle cell disease (SCD)	*HBB*	c.20 A > T (p.E6V or p.E7V)	HDR (IDLV donor)^28^
			HDR (AAV donor)^27,29^
			PE^96^
Tay-Sachs disease	*HEXA*	c.1274_1278dup	PE^96^
Xeroderma pigmentosum, complementation group C (XPC)	*XPC*	c.1840C > T (p.R579X)	ABE (CRISPR-Pass)^91^

*Adv* adenoviral vector, *IDLV* integrase-defective lentiviral vector, *AAV* adeno-associated viral vector, *PTC* premature termination codon.

^a^Inducing mutations that alleviate disease symptoms.

^b^Using the normal allele on the homologous chromosome as a template.

On the other hand, CRISPR-based base editors, which enable precise, highly efficient base conversion without requiring donor DNA or producing DNA cleavage, represent an alternative approach. However, CBEs and ABEs can convert bystander bases positioned within the editing window as well as target bases, generating unwanted mutations^74,75^ and, in such cases, failing to produce exact gene corrections. Furthermore, these tools are limited in the types of targeted base conversions they induce (C ∙ G→T ∙ A or A ∙ T→ G ∙ C only). In addition, several recent studies reported genome-wide off-target deaminase effects on DNA (for CBEs)^99,100^ and RNA (for CBEs and ABEs)^101,102^ and unwanted effects on DNA cytosines (for ABEs)^103^, necessitating further improvement. The recently developed CAST and INTEGRATE methods represent alternative gene knockin strategies, but their use has not yet been demonstrated in mammalian cells. PE is likewise a potentially attractive tool that requires further characterization.

In summary, all gene-editing tools are associated with some limitations or side effects, such as off-target editing, but intensive research has led to rapid improvement to compensate for these limitations. In the near future, we anticipate that several genetic diseases that lack reliable drugs will be treatable with an appropriate gene recovery strategy on the basis of the underlying genomic abnormality. Such precise endogenous gene recovery will herald a new era in human genetic medicine.
